# Impact of Pre-injury Statin Use on Mortality and Short-Term Clinical Outcomes Following Intracerebral Hemorrhage: A Propensity-Matched Cohort Study

**DOI:** 10.7759/cureus.113251

**Published:** 2026-07-23

**Authors:** Katelyn A Robertson, Kaitlyn Blake, Ashley Thompson, Rejoice Spivey, Tyler Thompson, Mrinalini Deverapalli, Miriam Michael

**Affiliations:** 1 Neurosurgery, Howard University Hospital, Washington, DC, USA; 2 College of Medicine, Howard University College of Medicine, Washington, DC, USA; 3 Internal Medicine, Howard University Hospital, Washington, DC, USA; 4 Radiology, Howard University College of Medicine, Washington, DC, USA; 5 Neurological Surgery, Howard University College of Medicine, Washington, DC, USA

**Keywords:** cohort study, intracerebral hemorrhage, mortality, neurocritical care, propensity score matching, statins, stroke outcomes

## Abstract

Background

Intracerebral hemorrhage (ICH) is associated with high mortality and limited therapeutic options. The impact of statin therapy on outcomes following ICH remains controversial.

Methods

A retrospective cohort study was conducted using the TriNetX Global Collaborative Network. Adult patients (≥18 years) with nontraumatic ICH were identified and stratified based on statin use within three months prior to the index event. Propensity score matching (1:1) was performed to balance demographics, comorbidities, and medication use. The primary outcome was 30-day mortality. Secondary outcomes included ICU admission or mechanical ventilation, external ventricular drain (EVD) placement, seizures, and craniotomy or shunt procedures.

Results

After matching, 82,755 patients were included in each cohort. Pre-injury statin use was associated with significantly lower mortality (13.4% vs 19.7%; risk ratio (RR) 0.68, 95% CI 0.66-0.70, p<0.001). Statin use was also associated with lower rates of ICU admission or mechanical ventilation (10.9% vs 13.7%; RR 0.80, p<0.001) and EVD placement (4.0% vs 4.6%; RR 0.87, p<0.001). Rates of seizures were similar between groups. Statin users had slightly higher rates of craniotomy or shunt procedures (0.8% vs 0.6%; RR 1.33, p<0.001).

Conclusion

Pre-injury statin use was associated with reduced mortality and improved short-term outcomes following intracerebral hemorrhage. These findings demonstrate an association between pre-injury statin use and lower mortality and differences in short-term clinical outcomes following intracerebral hemorrhage. Given the retrospective observational design, causality cannot be established.

## Introduction

Intracerebral hemorrhage (ICH) accounts for approximately 10-15% of all strokes but contributes disproportionately to stroke-related mortality and disability [1]. Despite advances in neurocritical care, outcomes remain poor, and effective therapeutic interventions are limited.

Statins are widely prescribed for the prevention of cardiovascular and cerebrovascular disease. Beyond their lipid-lowering effects, statins exhibit pleiotropic properties, including anti-inflammatory, antioxidative, and endothelial-stabilizing effects, which may influence neurological outcomes [2,3]. However, their role following ICH remains controversial. While some studies have reported an association between statin use and improved survival or functional outcomes, concerns persist regarding a potential increased risk of hemorrhage and hematoma expansion [4,5].

Given these conflicting findings, further investigation using large, real-world datasets is warranted. Using a large multicenter electronic health record database, this retrospective observational study evaluated the association between pre-injury statin use and short-term clinical outcomes following ICH.

## Materials and methods

Study design and data source

This retrospective cohort study was conducted using the TriNetX Global Collaborative Network, a federated database containing de-identified electronic health records from over 170 healthcare organizations. The network includes data on patient demographics, diagnoses, procedures, medications, and healthcare utilization collected from participating institutions in real time.

Cohort selection

Adult patients (≥18 years) with a diagnosis of nontraumatic intracerebral hemorrhage (ICD-10 code I61) were identified [6]. Patients with traumatic intracranial injury were excluded.

Patients were stratified into two cohorts based on documented statin prescriptions within three months prior to the index hemorrhage event. The statin cohort included patients with documented prescriptions for statins, including atorvastatin, simvastatin, or rosuvastatin. The non-statin cohort included patients without documented statin prescriptions during this period. Because TriNetX records prescription data rather than pharmacy dispensing or medication adherence, treatment exposure was defined by documented prescription history, and actual medication adherence or persistence could not be confirmed.

Propensity score matching

One-to-one propensity score matching was performed to reduce measured confounding. Covariates included age, sex, race, and comorbidities such as hypertension, diabetes mellitus, atrial fibrillation, chronic kidney disease, and cardiovascular disease, as well as concurrent use of anticoagulant and antiplatelet medications.

After matching, balanced cohorts of 82,755 patients in each group were achieved. Standardized mean differences were less than 0.1 for all included variables, indicating adequate balance between cohorts. Clinical and radiographic variables including admission neurological severity (e.g., Glasgow Coma Scale and NIH Stroke Scale scores), hematoma volume, hemorrhage location, intraventricular extension, hematoma expansion, and ICH score were not available within the TriNetX database and therefore could not be incorporated into the matching process.

Outcomes

Outcomes were assessed within 30 days of the index intracerebral hemorrhage event. The primary outcome was all-cause mortality. Secondary outcomes included craniotomy or cerebrospinal fluid diversion procedures, seizure occurrence, intensive care unit admission, requirement for mechanical ventilation, and external ventricular drain placement. Functional neurological outcome measures, including modified Rankin Scale and Barthel Index scores, were not available within the database and therefore were not evaluated.

Statistical analysis

Risk differences, risk ratios, and odds ratios were calculated for all outcomes. Kaplan-Meier survival analysis was performed for mortality, and comparisons between groups were made using the log-rank test. A p-value of less than 0.05 was considered statistically significant.

Ethical approval

The TriNetX Global Collaborative Network provides access to de-identified patient data; therefore, this study was exempt from institutional review board approval.

## Results

Baseline characteristics 

A total of 341,606 non-statin users and 118,239 statin users with ICH were identified in the initial cohort. Prior to matching, statin users were older and had a higher prevalence of comorbidities, including hypertension, diabetes mellitus, atrial fibrillation, chronic kidney disease, and prior cerebrovascular disease. Statin users were also more likely to be receiving antiplatelet and anticoagulant therapy.

After 1:1 propensity score matching, 82,755 patients remained in each cohort. Baseline characteristics were well-balanced between groups, with standardized mean differences <0.1 across all measured covariates, indicating adequate balance for the variables included in the matching process (Table 1). Clinical and radiographic measures of ICH severity, including hematoma volume, hemorrhage location, admission neurological severity, and ICH score, were not available within the database and therefore were not included in the matching process.

**Table 1:** Baseline characteristics before and after propensity score matching Abbreviations: SD, standard deviation Note: Propensity score matching was performed using demographics, comorbidities, and medication use. Statistical comparisons were performed using independent samples t-tests for continuous variables and chi-square tests for categorical variables. After matching, standardized mean differences for all covariates were <0.1, indicating adequate balance between cohorts.

Characteristic	Before Matching Non-Statin (n=270,115)	Before Matching Statin (n=105,479)	After Matching Non-Statin (n=82,755)	After Matching Statin (n=82,755)
Age (years)	62.6 ± 18.1	67.9 ± 13.1	68.1 ± 14.1	67.2 ± 13.3
Female, n (%)	123,829 (48.5)	47,439 (45.1)	38,730 (46.8)	38,280 (46.3)
Male, n (%)	131,240 (51.4)	57,682 (54.8)	43,964 (53.1)	44,416 (53.7)
White, n (%)	117,320 (46.0)	65,244 (62.0)	50,879 (61.5)	50,083 (60.5)
Black, n (%)	26,880 (10.5)	17,537 (16.7)	12,610 (15.2)	12,672 (15.3)
Hypertension, n (%)	71,618 (28.1)	68,833 (65.4)	48,780 (58.9)	47,379 (57.3)
Diabetes mellitus, n (%)	26,911 (10.5)	34,399 (32.7)	21,158 (25.6)	21,064 (25.5)
Chronic kidney disease, n (%)	18,000 (7.1)	20,759 (19.7)	12,705 (15.4)	13,045 (15.8)
Atrial fibrillation, n (%)	19,609 (7.7)	21,962 (20.9)	13,519 (16.3)	13,864 (16.8)
Heart failure, n (%)	17,712 (6.9)	20,810 (19.8)	11,929 (14.4)	12,548 (15.2)
Prior stroke, n (%)	24,130 (9.5)	37,609 (35.8)	17,865 (21.6)	20,130 (24.3)
Aspirin use, n (%)	33,620 (13.2)	49,013 (46.6)	29,073 (35.1)	29,360 (35.5)
Clopidogrel use, n (%)	9,492 (3.7)	17,649 (16.8)	8,406 (10.2)	9,117 (11.0)
Warfarin use, n (%)	8,306 (3.3)	9,234 (8.8)	6,074 (7.3)	6,021 (7.3)
Apixaban use, n (%)	7,196 (2.8)	9,953 (9.5)	5,575 (6.7)	5,878 (7.1)

Primary outcome: mortality 

At 30 days following ICH, patients with pre-injury statin use had a significantly lower observed mortality than those without statin use (13.4% vs. 19.7%). This corresponded to an absolute risk difference of 6.2% (95% CI, 5.9-6.6). Pre-injury statin use was associated with a lower risk of death compared with no statin use (RR 0.68, 95% CI, 0.66-0.70; p<0.001).

**Figure 1:**
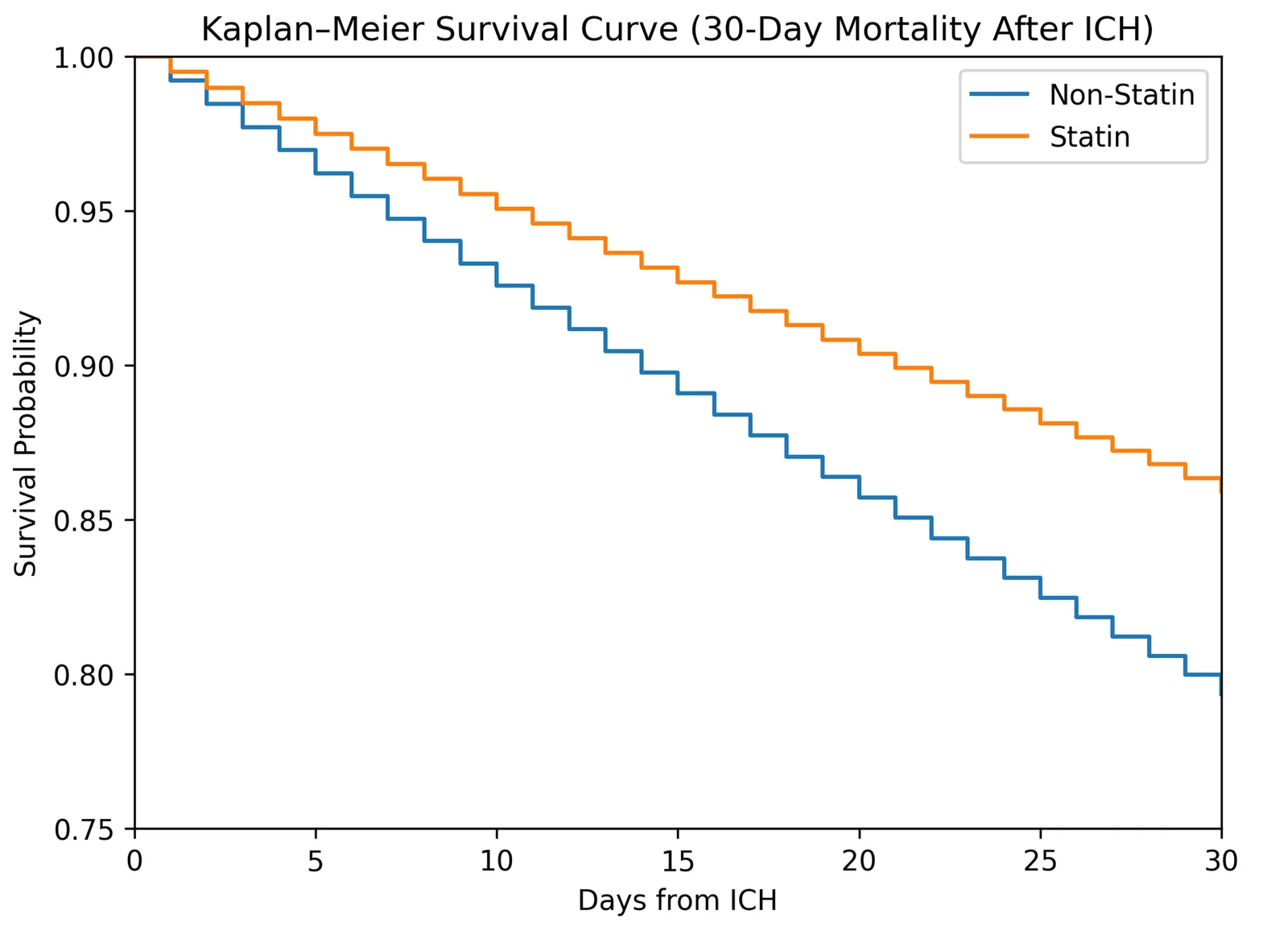
Kaplan–Meier survival curve comparing 30-day mortality between statin and non-statin cohorts following intracerebral hemorrhage The Kaplan-Meier analysis demonstrated significantly greater 30-day survival among patients with pre-injury statin use (log-rank p<0.001).

Kaplan-Meier survival analysis demonstrated significantly greater 30-day survival among patients with pre-injury statin use (Figure 1). The hazard ratio for mortality was 1.55 (95% CI, 1.51-1.59; log-rank p<0.001), indicating a higher observed mortality risk in the non-statin cohort during the follow-up period.

Secondary outcomes 

Thirty-day secondary clinical outcomes following propensity score matching are summarized in Table 2. Compared with the non-statin cohort, patients with pre-injury statin use had lower observed rates of ICU admission or mechanical ventilation and external ventricular drain (EVD) placement, while seizure incidence was similar between groups. Patients with pre-injury statin use also had a modestly higher observed rate of craniotomy or cerebrospinal fluid diversion procedures (Table 2).

**Table 2:** Thirty-day clinical outcomes after propensity score matching Statistical comparisons were performed using chi-square tests. Risk differences represent absolute differences between groups. Risk ratios less than 1 indicate lower risk among statin users compared to non-statin users.

Outcome	Non-Statin (n=82,755)	Statin (n=82,755)	Risk Difference (95% CI)	Risk Ratio (95% CI)	p-value
Mortality, n (%)	15,848 (19.7)	10,974 (13.4)	0.062 (0.059–0.066)	0.68 (0.66–0.70)	<0.001
Craniotomy/Shunt, n (%)	528 (0.6)	655 (0.8)	-0.002 (-0.002 to -0.001)	1.23 (1.11–1.39)	<0.001
Seizures, n (%)	3,588 (4.6)	3,439 (4.4)	0.002 (-0.000–0.004)	0.96 (0.92–1.01)	0.089
ICU Admission or Mechanical Ventilation, n (%)	10,854 (13.7)	8,332 (10.9)	0.028 (0.025–0.031)	0.80 (0.77–0.81)	<0.001
EVD Placement, n (%)	3,755 (4.6)	3,196 (4.0)	0.006 (0.004–0.008)	0.87 (0.83–0.91)	<0.001

Craniotomy or Cerebrospinal Fluid Diversion Procedures

Craniotomy or cerebrospinal fluid diversion procedures were performed slightly more frequently among patients with pre-injury statin use than among those without statin use (0.8% vs. 0.6%). This corresponded to a modest but statistically significant association with procedural intervention (RR 1.23, 95% CI, 1.11-1.39; p<0.001).

Seizures 

There was no statistically significant difference in seizure incidence between patients with and without pre-injury statin use (4.4% vs. 4.6%; RR 0.96, 95% CI, 0.92-1.01; p=0.089).

ICU Admission or Mechanical Ventilation

Patients with pre-injury statin use had lower observed rates of ICU admission or mechanical ventilation than those without statin use (10.9% vs. 13.7%). This corresponded to a lower observed relative risk (RR 0.80, 95% CI, 0.77-0.81; p<0.001).

External Ventricular Drain (EVD) Placement

EVD placement occurred less frequently among patients with pre-injury statin use than among those without statin use (4.0% vs. 4.6%). This corresponded to a lower observed relative risk (RR 0.87, 95% CI, 0.83-0.91; p<0.001).

Overall outcome comparison 

**Figure 2:**
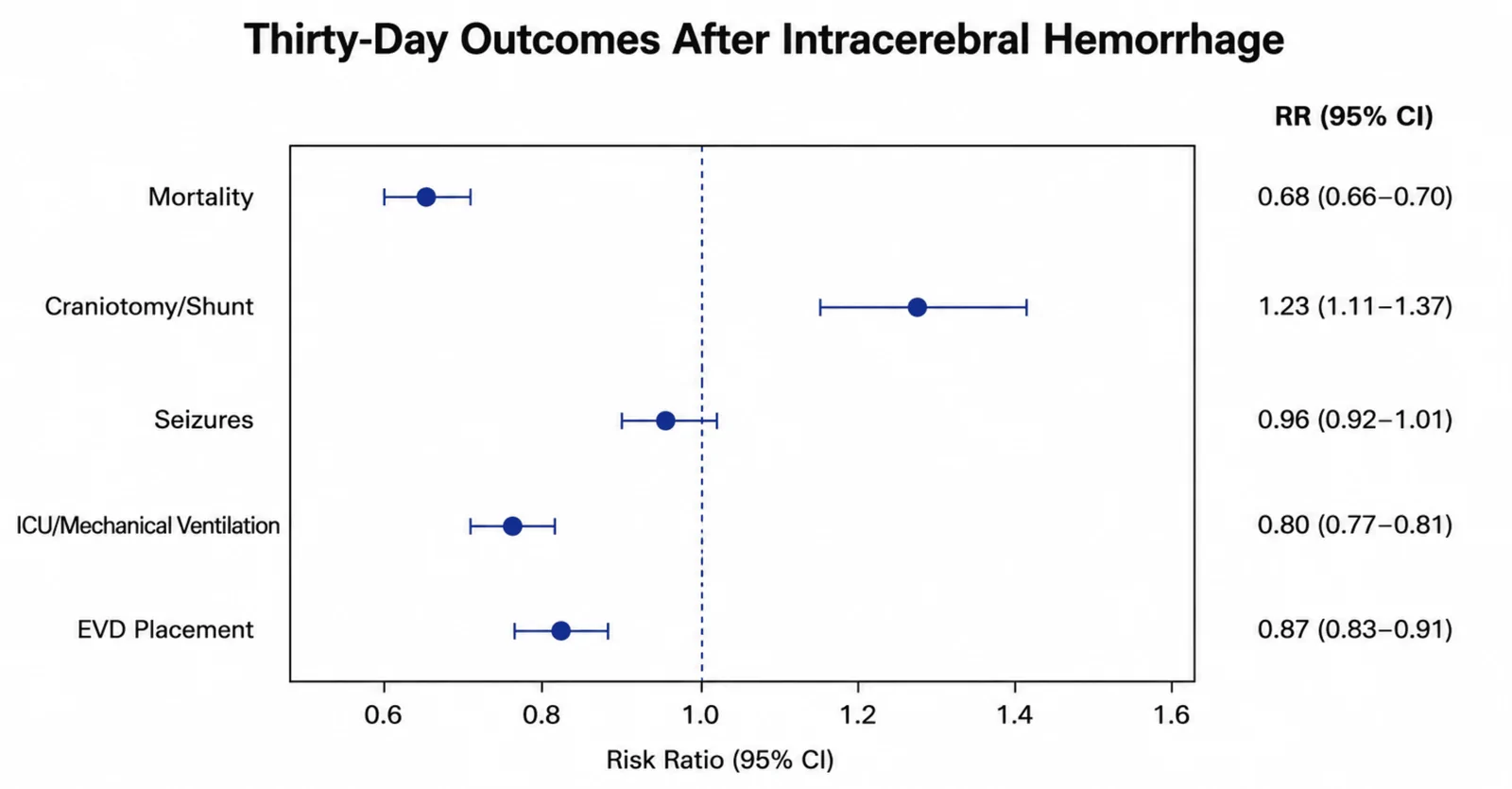
Forest plot of 30-day clinical outcomes following intracerebral hemorrhage comparing statin and non-statin cohorts Risk ratios (RRs) are presented with 95% confidence intervals. Values less than 1 indicate lower risk among statin users compared to non-statin users, while values greater than 1 indicate higher risk among statin users.

A forest plot summarizing the primary and secondary outcomes demonstrated a consistent pattern of associations between pre-injury statin use and the evaluated clinical outcomes, with the largest observed differences seen for 30-day mortality, ICU admission or mechanical ventilation, and external ventricular drain placement (Figure 2).

## Discussion

In this large, propensity score-matched retrospective cohort study, pre-injury statin use was associated with significantly lower 30-day mortality following ICH. Pre-injury statin use was also associated with lower observed rates of intensive care unit admission, mechanical ventilation, and external ventricular drain placement. Given the observational nature of this study, these findings should be interpreted as associations rather than evidence of a causal treatment effect.

These findings are consistent with prior observational studies reporting an association between statin use and improved outcomes following ICH [7,8]. Flint et al. reported lower in-hospital mortality and improved discharge disposition among patients maintained on statin therapy after ICH, while Tapia-Pérez et al. reported improved neurological recovery among patients who continued statin therapy following spontaneous hemorrhage [7,8]. Previous studies have also reported worse neurological outcomes following statin discontinuation, further supporting the need for continued investigation of statin therapy in cerebrovascular disease [9].

Although previous observational studies have reported an association between statin use and improved survival after ICH, many were limited by single-center designs or relatively small sample sizes. In contrast, the present study analyzed more than 165,000 propensity score-matched patients from a multinational electronic health record network, providing a large and diverse cohort. Propensity score matching achieved excellent balance across measured demographic characteristics, cardiovascular comorbidities, and concomitant medication use. However, despite adjustment for measured confounders, important determinants of ICH prognosis, including hematoma volume, hemorrhage location, intraventricular extension, admission neurological severity (GCS and NIHSS), ICH score, hematoma expansion, and surgical timing, were unavailable within the TriNetX database and therefore could not be incorporated into the matching process. Consequently, residual confounding remains possible, and the observed associations should be interpreted with appropriate caution. These prognostic factors are well established in the assessment and management of ICH and are incorporated into contemporary clinical frameworks and practice guidelines [10,11].

The observed association between pre-injury statin use and lower mortality is comparable to findings reported in previous retrospective analyses of patients with ICH. Our study expands upon the existing literature through the use of a substantially larger multicenter cohort and rigorous propensity score matching methodology, allowing for improved adjustment of measured baseline differences between cohorts. Nevertheless, because of the retrospective design and the potential for residual confounding, these findings should not be interpreted as demonstrating an independent mortality benefit attributable to statin therapy.

Several biological mechanisms may plausibly explain the observed associations. Beyond their lipid-lowering effects, statins possess anti-inflammatory, antioxidant, immunomodulatory, and endothelial-stabilizing properties that may attenuate secondary brain injury following ICH. Experimental and translational studies have demonstrated that statins reduce inflammatory cytokine production, preserve blood-brain barrier integrity, improve endothelial nitric oxide synthase activity, reduce oxidative stress, and enhance cerebral microvascular perfusion [2,3]. These effects may mitigate perihematomal edema, limit neuronal apoptosis, and improve cerebral autoregulation during the acute phase of hemorrhagic stroke. Statins have also been associated with enhanced angiogenesis, neurogenesis, and synaptic remodeling during recovery. While these proposed mechanisms provide biological plausibility for the observed associations, they do not establish causality and require confirmation in prospective clinical studies.

Patients with pre-injury statin use demonstrated a modestly higher rate of craniotomy or cerebrospinal fluid diversion procedures. Several explanations may account for this finding. Survivorship bias may have contributed, as patients surviving the acute phase of hemorrhage are more likely to undergo neurosurgical intervention. Alternatively, unmeasured differences in hemorrhage characteristics, including hematoma size, location, surgical accessibility, or other radiographic features unavailable within the database, may have influenced operative decision-making. Because these variables were not captured within TriNetX, the mechanism underlying this association cannot be determined.

No significant difference in seizure incidence was observed between groups, suggesting that pre-injury statin use was not associated with seizure occurrence within 30 days following ICH.

Clinical implications

Pre-injury statin use was associated with lower 30-day mortality and differences in several short-term clinical outcomes following ICH. However, these findings should be interpreted within the context of a retrospective observational study and do not establish a causal relationship between statin exposure and improved outcomes. Prospective studies incorporating detailed clinical, radiographic, and functional outcome data are needed to determine whether these observed associations represent a true therapeutic effect.

Limitations

This study has several important limitations. First, as a retrospective observational analysis, it is inherently susceptible to residual confounding despite propensity score matching. Although matching achieved excellent balance across measured covariates, several important predictors of ICH prognosis, including hematoma volume, hemorrhage location, intraventricular extension, perihematomal edema, hematoma expansion, admission neurological severity (GCS and NIHSS), ICH score, anticoagulation status at presentation, early neurological deterioration, and surgical timing, were unavailable within the TriNetX database and therefore could not be incorporated into the analyses.

Second, statin exposure was determined using documented prescription records within three months prior to the index hemorrhage. Medication adherence, pharmacy refill history, prescription persistence, cumulative statin exposure, statin intensity, and lipophilicity were not available and therefore could not be evaluated, introducing the potential for exposure misclassification.

Third, patients prescribed statins before ICH may differ systematically from non-users in ways that were not fully captured within the database. Healthy-user bias, differences in healthcare access, medication adherence, and other unmeasured factors may have contributed to the observed associations despite propensity score matching.

Fourth, secondary outcomes, such as intensive care unit admission, mechanical ventilation, external ventricular drain placement, and neurosurgical intervention, may be influenced by institutional practice patterns and provider decision-making in addition to disease severity. These outcomes should therefore be interpreted cautiously.

Finally, functional neurological outcome measures, including modified Rankin Scale, Barthel Index, discharge disposition, and long-term disability, were unavailable within the database, limiting assessment of long-term neurological recovery. Additional sensitivity analyses, including inverse probability weighting, multivariable Cox regression, and E-value analyses, as well as subgroup analyses according to statin type, intensity, lipophilicity, and cumulative exposure, were beyond the scope of the available dataset and represent important areas for future investigation. Despite these limitations, the large multicenter cohort and rigorous propensity score matching provide valuable real-world evidence describing the association between pre-injury statin use and short-term clinical outcomes following ICH.

## Conclusions

Pre-injury statin use was associated with lower 30-day mortality and differences in several short-term clinical outcomes following ICH, including lower observed rates of intensive care unit admission, mechanical ventilation, and external ventricular drain placement. Given the retrospective observational design of this study, these findings should be interpreted as associations rather than evidence of a causal treatment effect, and residual confounding cannot be excluded. Prospective studies incorporating detailed clinical, radiographic, and functional outcome measures are needed to determine whether these observed associations represent a true therapeutic effect and to better define the role of statins in patients with ICH.
